# Uncovering potential drug targets for lactylation-related genes mediated intervertebral disc degeneration: A Mendelian randomization analysis integrating eQTL data

**DOI:** 10.1097/MD.0000000000044382

**Published:** 2025-09-12

**Authors:** Yang Yang, Dingxuan Liu, Zhen Ai, Xi Gao

**Affiliations:** a Heilongjiang University of Chinese Medicine, Heilongjiang, China; b First Hospital of Heilongjiang University of Chinese Medicine, Heilongjiang, China.

**Keywords:** intervertebral disc degeneration, lactylation, Mendelian randomization, NDUFA13, NFU1

## Abstract

Intervertebral disc degeneration (IVDD) is a common cause of neck, back, and low back pain, it is also a major global public health problem. Lactate is a metabolic product of intervertebral disc cells and is closely related to disc degeneration. Lactylation (Lac), driven by lactic acid, is associated with intervertebral disc degeneration, and targeting-related genes may provide new therapeutic directions for intervertebral disc degeneration. In this study, data from genome-wide association studies were combined with drugs associated with Lac accumulation and expression quantitative trait loci (eQTLs) associated with Lac to explore the relationship between Lac-related genes and disc degeneration. Lac-related genes from MSigDB, intervertebral disc degeneration genome-wide association studies data from FinnGen, and Cis-eQTL data from the eQTLGen consortium. The inverse variance weighting (IVW) method was primarily used for evaluation, and sensitivity analyses were conducted using weighted median, simple mode, and weighted mode, which supported the robustness of results consistent with the IVW direction. The Cochran Q test assessed instrumental variable heterogeneity (*P* > .05), and a posterior probability PP.H4 > 0.80 was considered strong evidence of co-localization. A total of 36 potential genes reached statistical significance (*P* < .05) in the IVW method, but 23 genes in all sensitivity analysis methods had the same effect direction as IVW, demonstrating high robustness. Bayesian co-localization analysis identified NFU1 (PP.H4 = 1.00, OR = 1.18 [1.13–1.24]) and NDUFA13 (PP.H4 = 0.90, OR = 0.81 [0.74–0.89]), indicating that these 2 genes share causal variants associated with the risk of IVDD. NFU1 is a strong risk factor for IVDD, so the main type of drug selected is an “inhibitor.” NDUFA13 has a protective effect against IVDD, so the type of drug selected is an “agonist.” There is a causal association between Lac and IVDD, and IVDD progression can be delayed by regulating the expression of Lac-related druggable genes.

## 1. Introduction

The intervertebral disc (IVD) is a fibrous cartilage structure composed of the peripheral annulus fibrosus (AF), central nucleus pulposus (NP), the cartilaginous endplates on the upper and lower surfaces, and the extracellular matrix (ECM). It connects 2 adjacent vertebrae. The human spine contains 23 IVDs, which participate in movement and weight-bearing functions, exhibiting significant flexibility and the ability to withstand strong compressive, bending, and torsional loads.^[1]^ As aging occurs, IVD undergoes degeneration. Although IVDD is a normal part of aging, it often leads to changes in spinal anatomy, resulting in discomfort such as neck, back, and lower back pain.^[2]^ In addition, IVDD is more likely to occur in individuals with congenital spinal deformities, such as those with lumbar-sacral transition vertebrae (LSTV). The segment above the LSTV is more prone to IVDD, which is closely related to changes in spinal biomechanics. IVDD exacerbates the symptoms of low back pain, which is a leading cause of disability worldwide and has significant societal implications. Therefore, IVDD, as one of the most important causes of low back pain progression, has remained a hot topic of research.^[3]^

Recent studies have found that IVDD is becoming more prevalent in younger individuals. The causes of IVDD are complex and are usually closely related to injuries, but the main feature of IVDD is a degenerative cascade reaction associated with chronic inflammation, which is closely related to the metabolic homeostasis of the IVD.^[4,5]^ Research into targeted therapies related to IVDD metabolism is important.

IVD is a closed, anaerobic, avascular tissue due to its unique anatomical structure: the AF and the EP surround the NP. Therefore, a healthy IVD is an organ with special immune capabilities.^[6]^ The nutrition of the IVD mainly depends on the cellular metabolism within the IVD, so anaerobic glycolysis is the main energy supply method for the IVD, and NP cells are the main energy-supplying cells.^[1,4,7,8]^

The end product of anaerobic glycolysis is lactate, with this physiologic buildup of lactate over time the IVD is in an acidic environment. Despite the fact that the IVD in a healthy state is in an acidic environment, it has been found that the rate of proteoglycan synthesis is affected by the extracellular pH and is very sensitive.^[9]^ When lactate levels are elevated, the rate of proteoglycan synthesis is altered, which decreases proteoglycans and accelerating the onset of IVDD. At the onset of IVDD, decreased permeability of the EP results in decreased glucose and oxygen supply within the IVD, and stimulated by further hypoxia, glycolytic metabolism of NP cells is enhanced, resulting in increased lactate production, and a decrease in pH in this environment can lead to NP cell senescence and apoptosis, ECM imbalance and degradation, and accelerated development of IVDD.^[10–13]^

Therefore, elevated levels of lactate serve as a marker for the onset and progression of IVDD.^[7,13]^ This may be attributed to lactate accumulation promoting cell apoptosis and autophagy in NP while activating inflammatory pathways, ultimately accelerating the cascade of degenerative reactions in the IVD. In the past, lactate was merely regarded as a metabolic waste product. However, with increasing research, it has been discovered that lactate acts as a driving factor in the Lac process. Lac is a post-translational modification of proteins, occurring on lysine residues in proteins, including histones and non-histones.^[14]^

Lactate is thought to be a donor for lactoylation of histone lysine residues, which is ultimately involved in disease development by influencing transcriptional homeostasis.^[15]^ Since lactate levels are upregulated with the progression of IVDD, effective inhibition of Lac can be considered effective in mitigating the IVDD process, a view that has been confirmed by a recent study in which Zhang et al demonstrated that glutamine prevents IVDD through inhibition of glycolysis and reduction of AMPKα Lac, which in turn promotes autophagy and inhibits senescence in NP cells.^[5]^ These findings suggest that lactate and lactation are integral parts of the pathogenesis of IVDD and that targeting Lac may provide a potential strategy for preventing IVDD.

In this study, we aimed to integrate genome-wide association studies (GWAS) summary data on intervertebral disc degeneration with expression quantitative trait loci (eQTLs) associated with lactation using Mendelian randomization (MR) methods to elucidate the association between Lac and intervertebral disc degeneration. We also validated key genes through Bayesian co-localization analysis and identified drugs that have completed phase IV clinical trials, with the aim of providing new insights for the clinical treatment of intervertebral disc degeneration.

## 2. Methods

### 2.1. Study design

**Figure 1.**
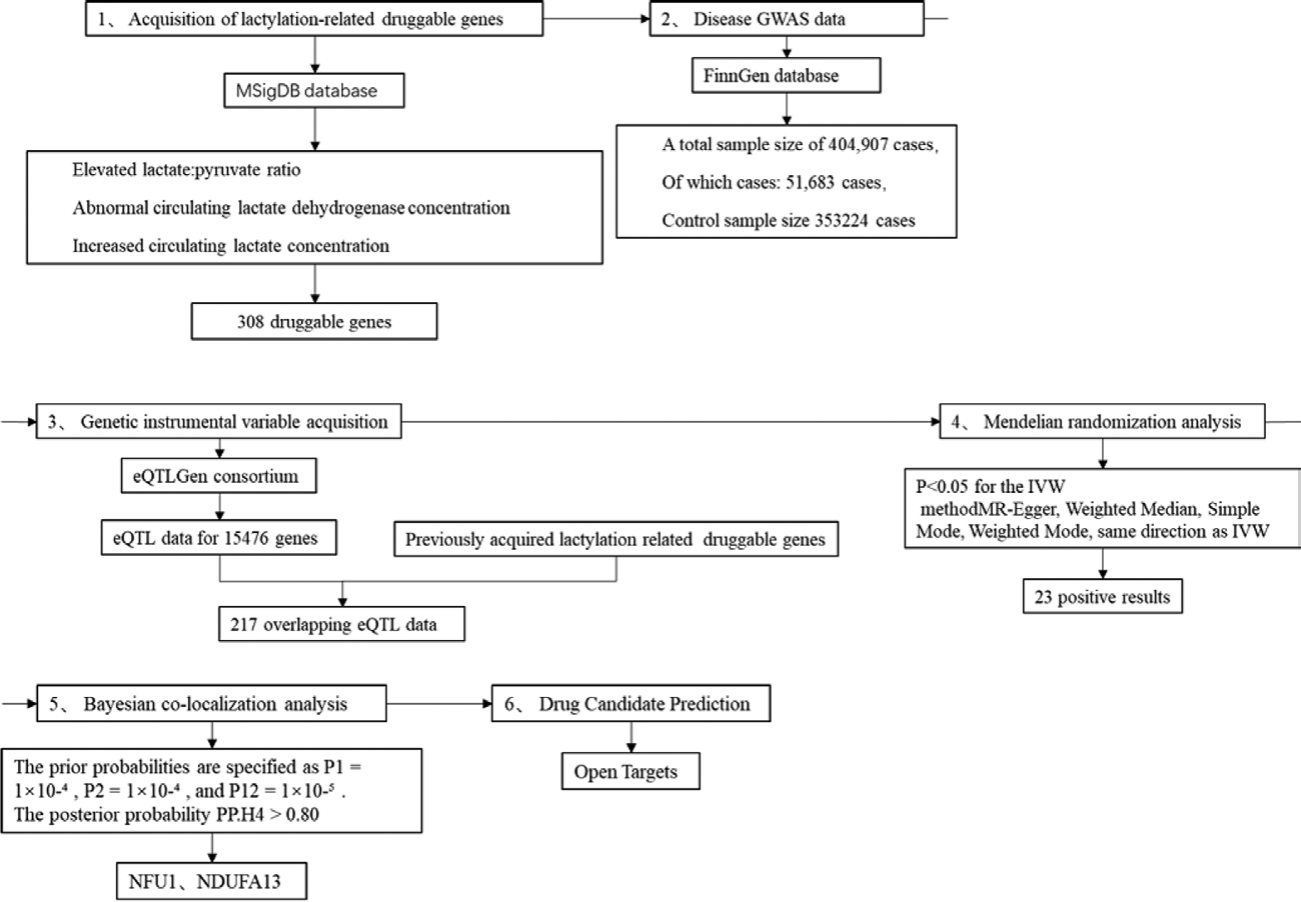
Study design flow. (1) Acquisition of lactylation-related genes from the MSigDB database, totaling 308 genes. (2) IVDD GWAS data obtained from the FinnGen database. (3) Cis-eQTL data obtained from the eQTLGen consortium, totaling 15,476, were used to identify genes overlapping with (1) acquisition of lactylation-related genes. (4) Mendelian randomization analysis. (5) Bayesian co-localization analysis. (6) Search for potential drug targets in Open Targets. eQTLs = expression quantitative trait loci, GWAS = genome-wide association studies, IVDD = intervertebral disc degeneration.

As shown in Figure 1, first, we included Lac-related druggable genes in the MSigDB database, and next, intersected with the cis-eQTL data obtained from the eQTLGen consortium to obtain the final eQTL instrumental variables (IVs) for this study, which were analyzed by MR analysis of the IVDD from the FinnGen database in order to identify potential druggable genes. To exclude the effect of linkage disequilibrium, we also performed Bayesian co-localization to obtain potential drug targets with strong association. To find drug candidates, we searched the Open Targets database for potential drug targets and looked for drugs that had completed phase IV clinical trials as candidates for IVDD treatment.

### 2.2. Data sources

#### 2.2.1. Druggable gene

Based on the fact that the conversion of pyruvate to lactate by lactate dehydrogenase is required for the formation of lactate during anaerobic glycolysis,^[16]^ we searched for “lactate” in the MSigDB database (https://www.gsea-msigdb.org/gsea/msigdb), and we retrieved 15 related gene files, and after screening, we finally included 3 gene files, which were “Elevated lactate: pyruvate ratio,” “Abnormal Elevated lactate: pyruvate ratio,” “Abnormal circulating lactate dehydrogenase concentration,” and “Increased circulating lactate concentration.” Ultimately, we included all non-redundant genes in these 3 gene sets, totaling 308 genes.

### 2.3. Summary statistics of GWAS used in this study

In this study, IVDD-associated GWAS data were used as the outcome data, which were obtained from the publicly available GWAS database: FinnGen database (https://www.finngen.fi/en/access_results), sample from “Other intervertebral disc disorders” (https://storage.googleapis.com/finngen-public-data-r12/summary_stats/release/finngen_R12_M13_INTERVERTEB.gz), with a total sample size of 404,907 cases included, of which the number of cases: 51,683, and the control sample size 353,224, all included sample size was of European origin.

### 2.4. Expression quantitative trait loci dataset for genetic IV selection

In this study, genetic variation acting “in cis” on the expression of drug-available genes (i.e., eQTLs) was used as exposing IV. First, cis-eQTL data (2019-12-11-cis-eQTLsFDR-ProbeLevel-CohortInfoRemoved-BonferroniAdded.txt.gz) and allele frequency data (2018-07-18_SNP_AF_for_AlleleB_combined_allele_counts_and_MAF_pos_added.txt.gz) were obtained in the eQTLGen consortium (https://eqtlgen.org/phase1.html). Next, the above 2 data were combined in the R package, and the effect allele frequency (eaf), effect size (beta), and standard error (SE) were calculated, and the *P*-value was set to *P* < 5e-8 to filter out the SNPs that were significantly associated with each other. We then removed the linkage disequilibrium (kb = 10000, *r*^2^ = 0.001) of the above SNPs and calculated the *F*-statistic by the following formula to filter out the rows with *F* ˃ 20 and remove the weak IVs.^[17]^ Finally, eQTL data of 15,476 genes were obtained.

Next, we screened the acquired 308 LRGS with the final 15,476 human cis-eQTL data acquired in the eQTLGen consortium to obtain 217 overlapping eQTL data, and used this dataset as the exposure for subsequent MR analysis. Screening of instrumental variables was required to ensure an accurate assessment of the causal effect between eQTL for Lac-related druggable genes and IVDD, and therefore, we screened IVs through the following steps: SNPs with *P* < 5e-8 were screened from IVDD-associated GWAS data, linkage disequilibrium were removed, and *r*² = 0.001, kb = 10,000 was set to ensure independence between IVs. To ensure the strength of SNPs in the IVs, we chose strong IVs with *F* > 10 to minimize the impact of weak IVs on MR analysis^[18]^ (*F* = (N - K - 1)/K*R^2^/(1 - *R*^2^)). Prior to the MR analysis, we performed multiple validity and heterogeneity tests to ensure the robustness and reliability of the MR assessment.

All data used in this study were obtained from publicly available databases and ethical approval and informed consent were obtained.

### 2.5. MR

MR analysis is a method of inferring the causal effect of exposure on outcome by using genetic variation as an instrumental variable. In the past, randomized controlled trial is often considered as the gold standard for evaluating the efficacy of drug therapy, but it is not fully feasible due to its high cost, long time consuming and ethical issues. MR is a method of causal inference based on genetic variation, which provides a theoretical basis for targeted therapies by using genetic factors as IVs to infer causal relationships between exposures and outcomes.

To ensure the reliability and accuracy of the results of this study, 3 important assumptions had to be fulfilled in this MR analysis: the instrumental variable is strongly associated with Lac-related druggable genes; the IV is independent of any confounders; and there is no horizontal pleiotropy, and the IV affects IVDD only through Lac-related druggable genes and not through any other potential pathway.

In this study, we mainly used inverse variance weighted (IVW) as a method to estimate the causal effect of LRGS and IVDD, and the results were considered statistically different if *P* < .05 for IVW method. We also used MR Egger to assess horizontal pleiotropy (significance threshold *P* > .05), and sensitivity analyses by weighted median, simple mode, and weighted mode, which further supported the robustness of the results when the above methods were in the same direction as IVW. In addition, we assessed instrumental variable heterogeneity by Cochran *Q* test (*P* > .05). For MR analysis, if there were missing SNPs, the data were obtained by converting “CHR” to “rsid” and manually searching “NCBI (https://www.ncbi.nlm.nih.gov)” to obtain the SNPs and excluded the data rows for which the SNPs could not be obtained in the end.

### 2.6. Bayesian co-localization analysis

Next, in this study, the above MR analysis was used to obtain positive result genes as exposures, eQTL data as IVs, and IVDD as the endpoint, and Bayesian co-localization analysis was performed to verify that the above MR positive genes were all driven by shared causal variants. We specified the prior probabilities as P1 = 1 × 10^-4^ (SNP associated with gene expression), P2 = 1 × 10^-4^ (SNP associated with IVDD), and P12 = 1 × 10^-5^ (SNP associated with 2 traits).^[19]^ The posterior probabilities of final association with outcome were derived from co-localization analyses as follows: for PPH0, not associated with Lac-related druggable genes expression or IVDD; for PPH1, associated with Lac-related druggable genes expression but not with IVDD; for PPH2, associated with outcome but not with Lac-related druggable genes expression; for PPH3, associated with LRGS expression and IVDD with different causal variants; for PPH4, associated with association of Lac-related druggable genes expression and IVDD with common causal variants. When the posterior probability PP.H4 > 0.80 was considered as strong evidence of co-localization, Lac-related druggable genes co-localized with IVDD were considered as potential drug targets.^[20]^

The aforementioned MR analysis and Bayesian co-localization analysis were both performed using R software (4.4.2), R Foundation for Statistical Computing, Vienna, Austria. MR mainly used the TwoSampleMR software package in R software, while Bayesian co-localization analysis mainly used the coloc software package.

### 2.7. Candidate drug prediction

Open Targets uses human genetics and genomics data for systematic drug target identification and prioritization as a freely available open-source tool that highlights variant-centric statistical evidence, allows prioritization of candidate causal variants at trait-associated loci, and identifies potential drug targets.^[21]^ We obtained investigated or approved drugs by submitting potential drug targets to the Open Targets database (https://www.opentargets.org/) to finalize the potential and therapeutic feasibility of potential dosing target genes associated with IVDD based on the riskiness and protectiveness of Lac-related druggable genes against IVDD.

## 3. Results

**Figure 2.**
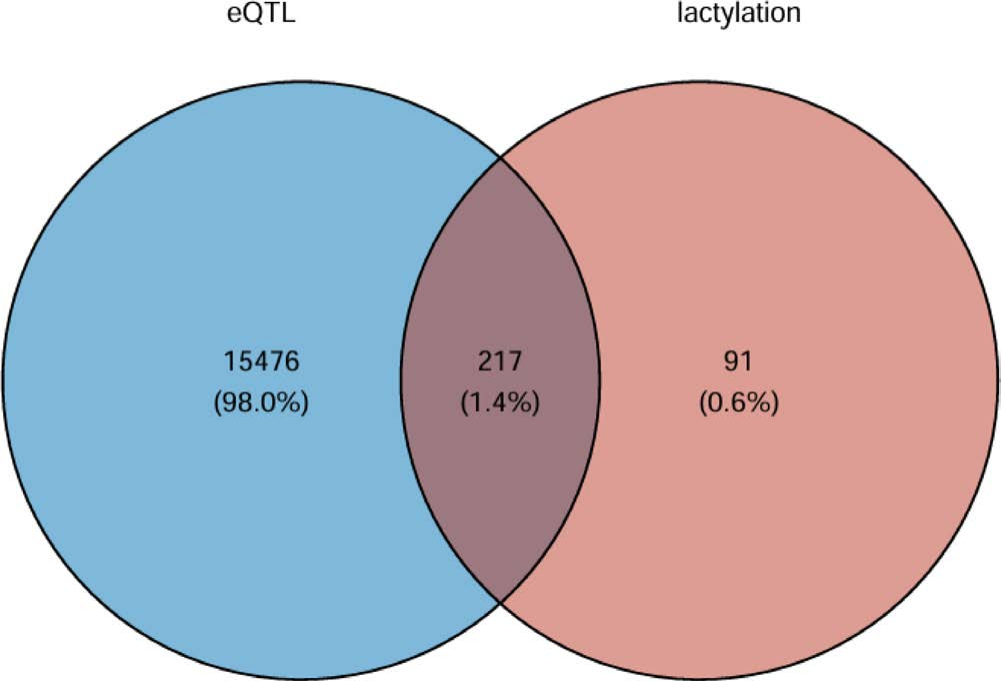
Venn diagram: overlap of eQTL and lactylation-related genes. (1) 15,693 genes in the eQTLGen consortium. (2) MSigDB database obtained 308 lactylation-related genes. (3) Overlapping gene 217. eQTLs = expression quantitative trait loci.

**Figure 3.**
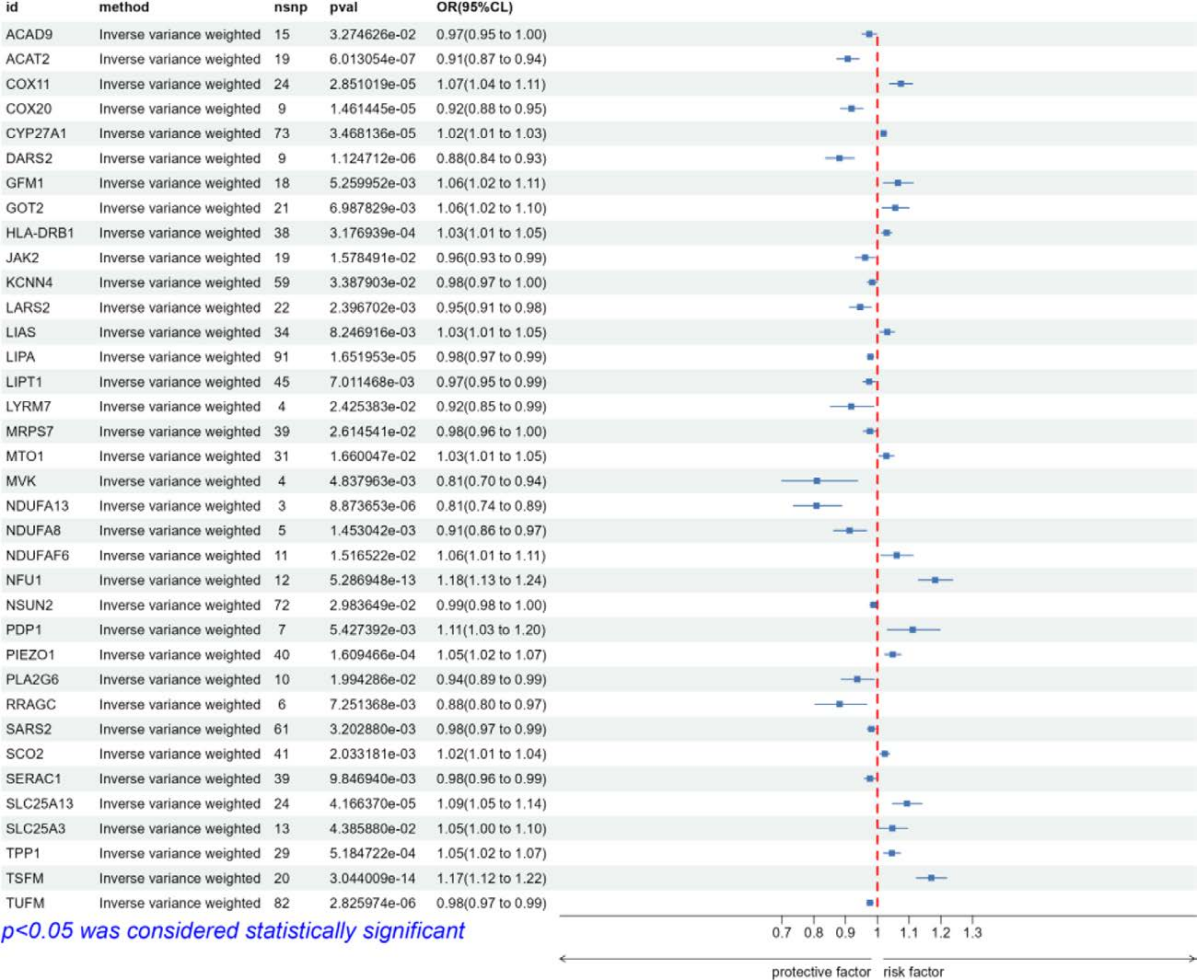
Forest plot of potential genes with consistent effect directions. IVW method, MR Egger, weighted median, simple model, and weighted model with *P* < .05 and consistent effect direction. An OR < 1 indicates that the gene has a protective effect against the disease, while an OR > 1 indicates that these genes pose a risk for the disease. IVW = inverse variance weighting, MR = Mendelian randomization.

As shown in Figure 2, we filtered the 308 LRGS with cis-eQTL data to obtain 217 overlapping eQTL data, and used this dataset as an IV for subsequent MR analysis. After MR analysis, the results of IVW method showed that a total of 36 potential genes were causally associated with IVDD (Fig. 3). All of the above genes reached statistical significance (*P* < .05) in the IVW method, but the direction of effect of all sensitivity analysis methods (MR Egger, weighted median, simple mode, and weighted mode) for 23 of these genes was completely consistent with IVW, and these 23 positive results had a high degree of robustness and were not significantly interfered with by horizontal multiplicity or aberrant SNPs. Among them, ACAD9, ACAT2, COX20, DARS2, JAK2, KCNN4, LARS2, LIPA, LIPT1, LYRM7, MRPS7, NDUFA13, NDUFA8, NSUN2, and PLA2G6 results showed OR < 1, indicating that these genes have strong protective effects against IVDD. While COX11, GFM1, HLA-DRB1, LIAS, MTO1, NDUFAF6, NFU1, and PIEZO1 results showed OR > 1, indicating that these genes are strongly risky for IVDD.

**Figure 4.**
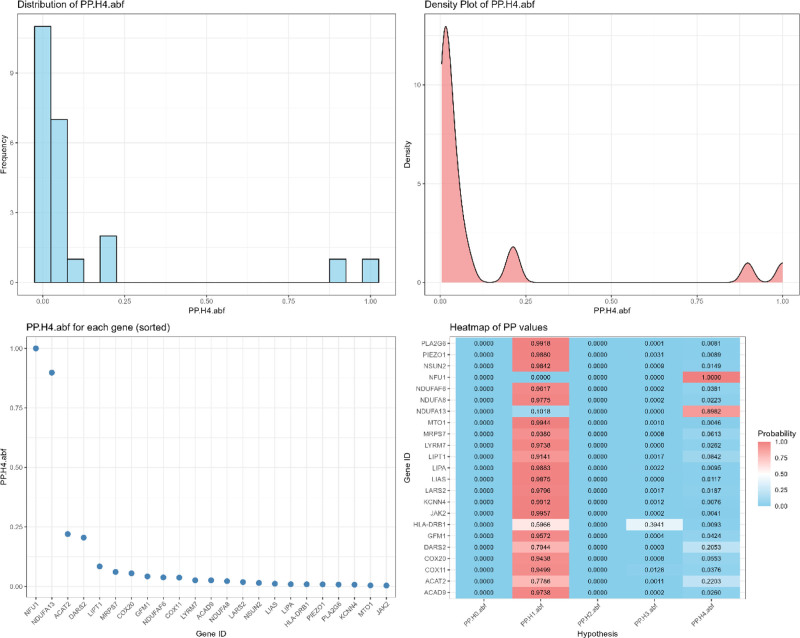
Bayesian co-localization analysis. PP.H4 > 0.80 proves that the 2 share the same causal variation and co-localization exists.

Subsequently, we performed a Bayesian co-localization analysis with IVDD after using the above 23 positive results with high robustness to verify whether the obtained associations were driven by shared causal variants. As shown in the results of Figure 4, a total of 2 genes had PP.H4 > 0.80, NFU1 (PP.H4 = 1.00, OR = 1.18 [1.13–1.24]) and NDUFA13 (PP.H4 = 0.90, OR = 0.81 [0.74–0.89]), suggesting that these 2 genes share a causal variant with the risk of IVDD, which further supports their role as therapeutic genetic evidence for the target. Since all of these genes are involved in mitochondrial energy metabolism, it suggests that disc degeneration may be closely related to imbalance of cellular energy homeostasis,^[22–25]^ NFU1 and NDUFA13 are both involved in mitochondrial energy metabolism pathway, NFU1 affects respiratory chain complex I function through iron-sulfur cluster (Fe-S) assembly and participates in mitochondrial redox reactions, while NDUFA13 (also known as GRIM-19) as the complex I subunit, may delay IVDD progression by regulating redox homeostasis with inhibition of inflammatory factors (e.g., IL-6, TNF-α).

Finally, we searched the potential drug targets, NFU1, NDUFA13, obtained from MR analysis and Bayesian co-localization analysis in the Open Targets database. Because NFU1 is a strong risk for IVDD, the drug type included is mainly “Inhibitor,” and NDUFA13 is protective against IVDD, so the drug type included is “Agonist.” Among the relevant drugs that finally completed the phase IV clinical trials, we obtained 13 drugs that inhibit NFU1 and 3 drugs that activate NDUFA13 expression (as shown in Table 1), among which CELECOXIB, DICLOFENAC as cyclooxygenase inhibitor are now widely used in the clinic for patients with low back pain, which is in line with the main IVDD clinical manifestations of IVDD.

**Table 1:** Drugs that have completed phase IV clinical trials.

Gene	Drug	Mechanism of action	Action type
NFU1	Methotrexate	Dihydrofolate reductase inhibitor	Inhibitor
	Apremilast	Phosphodiesterase 4 inhibitor	Inhibitor
	Lidocaine	Sodium channel alpha subunit blocker	Blocker
	Diclofenac	Cyclooxygenase inhibitor	Inhibitor
	Sulfasalazine	Cyclooxygenase inhibitor	Inhibitor
	Infliximab	TNF-alpha inhibitor	Inhibitor
	Colchicine	Tubulin inhibitor	Inhibitor
	Diclofenac potassium	Cyclooxygenase inhibitor	Inhibitor
	Adalimumab	TNF-alpha inhibitor	Inhibitor
	Celecoxib	Cyclooxygenase-2 inhibitor	Inhibitor
	Cytarabine	DNA polymerase (alpha/delta/epsilon) inhibitor	Inhibitor
	Certolizumab pegol	TNF-alpha inhibitor	Inhibitor
	Dasatinib	Stem cell growth factor receptor inhibitor	Inhibitor
NDUFA13	Methylprednisolone	Glucocorticoid receptor agonist	Agonist
	Dexamethasone	Glucocorticoid receptor agonist	Agonist
	Triamcinolone acetonide	Glucocorticoid receptor agonist	Agonist

## 4. Discussion

As the largest avascular tissue in the human body, the IVD primarily relies on anaerobic glycolysis as its main source of energy. Therefore, the IVD is constantly exposed to an acidic environment. IVDD is an age-related disease that occurs naturally with aging, but various factors that cause biomechanical changes are important factors that accelerate the onset of IVDD. Research has found that patients with congenital spinal deformities, such as LSTV, experience fusion of the affected segments, which protects the intervertebral discs from degeneration. However, due to changes in the biomechanics of adjacent intervertebral discs, the IVD above the lumbosacral transition vertebrae undergo accelerated degeneration.^[26–29]^ Communication between IVD and vertebral bodies primarily occurs through the permeability of the endplates. Research has shown that as vertebral maturity increases, the permeability of the endplates decreases, further reducing oxygen levels within the IVD and increasing lactate production from nucleus pulposus cell metabolism.^[30]^

Driven by various factors, mitochondrial endoplasmic reticulum stress and excessive production of reactive oxygen species induce aging-related phenotypes, activating and accelerating the aging of nucleus pulposus cells (NPC).^[31]^ When IVD is subjected to uneven forces, its closed environment is disrupted, leading to impaired immunity. Macrophages enter the IVD, triggering M1 macrophage polarization and infiltration associated with inflammation. This process activates the p38MAPK pathway associated with aging through the secretion of IL-6 and TNF-α, and upregulates the expression of aging markers such as p16INK4a.^[32]^ Biomechanical changes caused by various factors stimulate changes in the internal environment of the IVD, leading to metabolic imbalance and ultimately accelerating the aging of the NPC.^[31,33]^ NPCs are the primary functional units responsible for maintaining the stability of the IVD internal environment. NPC aging is an important mechanism in the development of intervertebral disc degeneration, and the accumulation of lactate further reduces NPC activity, inducing NPC apoptosis and accelerating the progression of IVDD.^[34,35]^

In addition, lactate post-translational modification is equally important for other orthopedic diseases, and it has been found that elevated levels of lactate and Lac can be detected in the peripheral blood of rheumatoid arthritis, which is one of the potential biomarkers for early diagnosis of rheumatoid arthritis and disease activity.^[36]^ The Lac genes are likewise an emerging target for osteoarthritis treatment.^[37]^ Although in recent years, the role of Lac in regulating the progression of various diseases and cells has received extensive attention in recent years, and with the finding that lactate metabolism is involved in the process of IVDD, the existence of a causal association with IVDD has not been confirmed, and there has not yet been a clear clinical indication that drugs are associated with the modulation of Lac-related genes in ameliorating IVDD.^[13,34,38]^

In this study, we revealed the causal relationship between NFU1, NDUFA13, and IVDD, and validated this causal effect through Bayesian co-localization analysis. Research has found that NFU1 is an iron-sulfur cluster scaffold protein expressed in mitochondria, responsible for assembling and transferring iron-sulfur clusters to target proteins, participating in the increase of pyruvate dehydrogenase, and participating in mitochondrial aerobic respiration.^[39]^ The NFU1 gene is located in the 2p13-p15 chromosomal region and is involved in encoding the NFU1 protein. Two NFU1 monomers assemble into an unstable 4Fe-4S cluster, which is prone to mutations that affect the mitochondrial respiratory chain.^[23]^ Therefore, suppressing the expression of this gene helps control its mutation and prevents anaerobic glycolysis from producing lactate. NDUFA13, also known as GRIM-19, is nicotinamide adenine dinucleotide dehydrogenase (quinone) 1 α subunit complex 13, a fundamental subunit of mitochondrial respiratory chain complex I. It is associated with mitochondrial membrane potential, mitochondrial oxidative stress, apoptosis, and the regulation of multiple signaling pathways, and is involved in aerobic respiration. Therefore, promoting the expression of this gene helps reduce lactate production from anaerobic glycolysis.^[40–42]^

Confounding bias in traditional observational studies was excluded by MR, and co-localization analysis (PP.H4 > 0.80) further confirmed that the associations of NFU1 (protected gene, OR = 1.18, 95% CI: 1.13–1.24, PFDR < 0.05, PP.H4 > 0.80) and NDUFA13 with IVDD were driven by shared causal variants, clarifying the potential role of these genes in the pathogenesis of IVDD and suggesting that these genes are drug targets of potential therapeutic value. In addition, this study further explored inhibitors of NFU1 (e.g., celecoxib) and agonists of NDUFA13 (e.g., dexamethasone) by combining them with the Open Targets database, which provides a theoretical basis for targeting the regulation of Lac to delay IVDD.

This study enriches the correlation, confirming a causal association between Lac and IVDD and providing a drug target. Although the present study found that inhibition of NFU1 and activation of NDUFA13 can be used to treat IVDD, whether the same therapeutic effect on other orthopedic diseases remains to be verified. Although the relevant drug candidates proposed in this study have completed phase IV clinical trials, their specific regulatory mechanisms on lactate metabolism or mitochondrial function in IVDD have not yet been clarified. Subsequent studies are needed to validate the effects of the drugs on the expression of NFU1/NDUFA13, lactate levels, and ECM metabolism in cellular models (e.g., myeloid cells) of IVDD or in animal models to confirm their targeted therapeutic efficacy.

Although the MR method is a well-established and robust method for causal inference, limitations remain. The dataset used was predominantly a European population, and more ethnic groups need to be included in future studies to increase generalizability. The Lac-related genes we obtained were only 3 datasets from MSigDB database, largely narrowing the scope of the study. In addition, this study was limited to cis-eQTL data for Lac-related genes, whereas the potential impact of trans-eQTL data on IVDD has not been explored. Therefore the relationship between IVDD, NFU1, NDUFA13, and Lac needs to be further analyzed in larger studies. In addition, more experimental studies are necessary to functionally validate our findings.

## 5. Conclusion

There is a causal relationship between Lac and IVDD. Our research results indicate that the progression of IVDD can be delayed by regulating the expression of pharmacologically relevant genes associated with Lac. Future research can promote the clinical development of anti-IVDD drugs by experimentally verifying target mechanisms, expanding multi-ethnic data, and exploring multi-target synergistic effects.

## Acknowledgments

Acknowledge anyone who contributed towards the article for authorship.

## Author contributions

**Data curation:** Yang Yang.

**Formal analysis:** Zhen Ai.

**Software:** Dingxuan Liu.

**Supervision:** Xi Gao.

**Validation:** Yang Yang.

**Writing – original draft:** Yang Yang.

**Writing – review & editing:** Yang Yang, Xi Gao.
